# Late Parotid Recurrence of Hepatocellular Carcinoma Following Liver Transplantation: A Case Report

**DOI:** 10.1155/crhe/2965476

**Published:** 2025-12-18

**Authors:** Anthony Onde Morada, Caroline Kai Chen, Clifford Akateh, Michael R. Marvin, Anil Kotru

**Affiliations:** ^1^ Department of General Surgery, Geisinger Wyoming Valley Medical Center, Wilkes-Barre, 18702, Pennsylvania, USA; ^2^ Department of Liver and Kidney Transplant Surgery, Geisinger Medical Center, Danville, 17822, Pennsylvania, USA, geisinger.edu

## Abstract

Metastatic recurrence to the parotid gland following liver transplantation has not been previously reported. We describe a case of a 63‐year‐old man with hepatocellular carcinoma secondary to alcoholic cirrhosis who was treated with transarterial chemoembolization and microwave ablation, achieving radiographic downstaging within the Milan criteria. He underwent liver transplantation with an exception Model for End‐Stage Liver Disease score of 27. Explant pathology revealed moderately differentiated multifocal tumors with negative margins. He remained recurrence‐free for 31 months under protocol‐based surveillance. He then developed a facial mass; two core needle biopsies were nondiagnostic. Surgical resection confirmed moderately to poorly differentiated metastatic hepatocellular carcinoma involving the parotid gland and zygomatic arch. Tumor markers rose only modestly at the time of recurrence. Despite targeted therapy and radiation, the disease progressed, culminating in widespread metastasis and death 13 months after recurrence. Immunotherapy was deferred due to the risk of graft rejection, and conversion to an mTOR‐based regimen was not recommended. This case highlights the challenges in long‐term posttransplant surveillance, diagnostic limitations of core biopsy in atypical lesions, and systemic treatment constraints in immunosuppressed patients.

## 1. Introduction

Hepatocellular carcinoma (HCC) is the most common primary liver malignancy and a leading cause of cancer‐related mortality worldwide [1]. Liver transplantation offers curative potential for patients meeting established criteria such as the Milan or UCSF guidelines. Despite careful selection, recurrence occurs in 10%–20% of recipients, usually within two years [2].

Extrahepatic recurrence typically involves the lungs, bones, or lymph nodes. Head and neck metastases are rare [3, 4], with nasopharyngeal involvement reported in only isolated cases [5], and metastasis to the parotid gland has not been previously reported in transplant recipients. We describe a case of delayed parotid recurrence that highlights diagnostic delays, surveillance gaps, and treatment limitations in posttransplant oncology.

## 2. Case Presentation

A 63‐year‐old man with alcoholic cirrhosis was evaluated for liver transplantation in 2019 after the incidental detection of HCC. A year earlier, he had presented with hematemesis and was diagnosed with *Helicobacter pylori*–positive peptic ulcers, which were treated with triple therapy. MRI identified two LI‐RADS, 5 lesions in Segments 4A and 8, measuring 29 and 18 mm, respectively. AFP peaked at 7.8 ng/mL. He underwent three transarterial chemoembolization (TACE) sessions and one microwave ablation. By July 2019, imaging confirmed downstaging with a radiographic response within Milan criteria and normalization of AFP to less than 1.0 ng/mL.

He underwent orthotopic liver transplantation with an HCC exception MELD score of 27. The donor was a 13‐year‐old male after circulatory death. Cold and warm ischemia times were 2 h, 59 min, and 34 min, respectively. CMV status was donor‐positive, recipient‐negative. Induction immunosuppression included methylprednisolone sodium succinate, with mycophenolate mofetil and tacrolimus initiated on Postoperative day 2.

Explant pathology showed pT2, moderately differentiated multifocal HCC with negative margins, and no macrovascular invasion. The postoperative course was complicated by MRSA bacteremia, bile leak, and abdominal compartment syndrome, requiring three reoperations. A liver biopsy revealed mild acute cellular rejection (RAI 3). He was discharged on tacrolimus and mycophenolate, with valganciclovir for CMV prophylaxis.

The patient remained recurrence‐free during protocol‐based surveillance, including imaging at 3, 12, and 24 months and serial AFP testing. AFP remained < 1.1 ng/mL through 2022. At 31 months posttransplant, he developed left facial swelling. Imaging revealed a 3.8‐cm enhancing lesion in the left masseter muscle with erosion of the zygomatic arch (Figure 1). AFP had risen slightly to 1.8 ng/mL. Two core needle biopsies were nondiagnostic.

Figure 1Multimodal CT imaging of recurrent metastatic hepatocellular carcinoma in the zygomatic arch: (a) axial contrast–enhanced CT demonstrates a 7.0‐cm heterogeneously enhancing mass centered in the left zygomatic arch with aggressive local invasion into the left maxillary sinus, extraconal orbital fat, and masticator/parotid spaces. (b) Coronal view shows bony destruction of the temporomandibular joint articular fossa. The lesion exhibits preserved vascular enhancement, which is characteristic of hepatocellular carcinoma.

**Figure:**
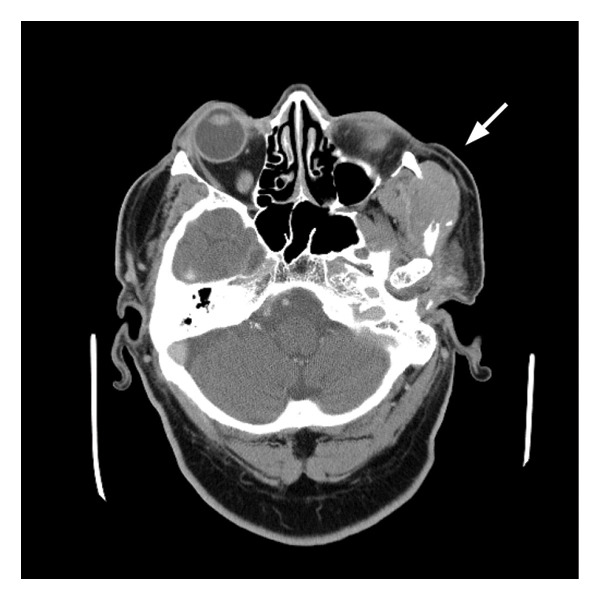
(a)

**Figure:**
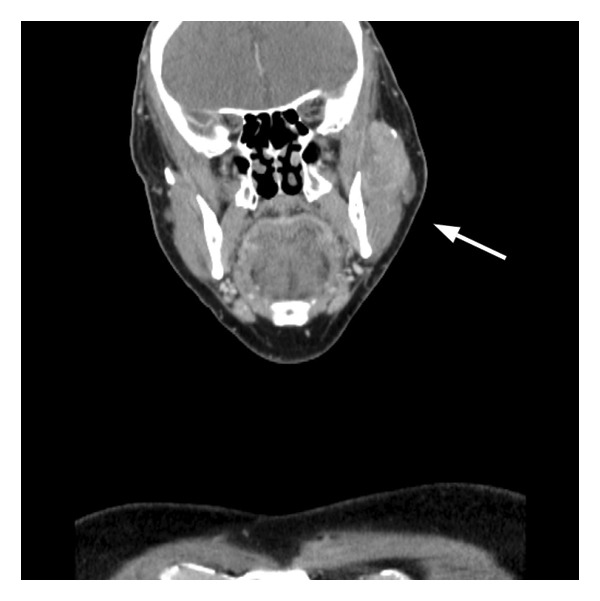
(b)

He underwent superficial parotidectomy and infratemporal tumor resection. Pathology confirmed metastatic, moderately to poorly differentiated HCC involving the parotid gland and adjacent soft tissue (Figure 2). The tumor stained positive for Arginase‐1 and Glypican‐3, with PD‐L1 expression of 2%–4%. It was microsatellite‐stable with a low tumor mutational burden (5.69). Postoperatively, AFP rose modestly to 4.0 ng/mL.

Figure 2Histopathological features of metastatic hepatocellular carcinoma: (a) hematoxylin and eosin (H&E), 200x magnification, demonstrates a poorly differentiated HCC with pleomorphic vesicular nuclei, prominent nucleoli, and atypical mitotic figures. (b) H&E, 100x, highlights areas of coagulative necrosis bordering viable tumor nests, consistent with post‐TACE changes. (c) H&E, 40x, shows steatohepatitic differentiation with macrovesicular fat droplets, recapitulating hepatic morphology. (d) H&E, 20x, reveals focal infiltration of native parotid salivary tissue without complete parenchymal replacement, confirming metastatic spread.

**Figure:**
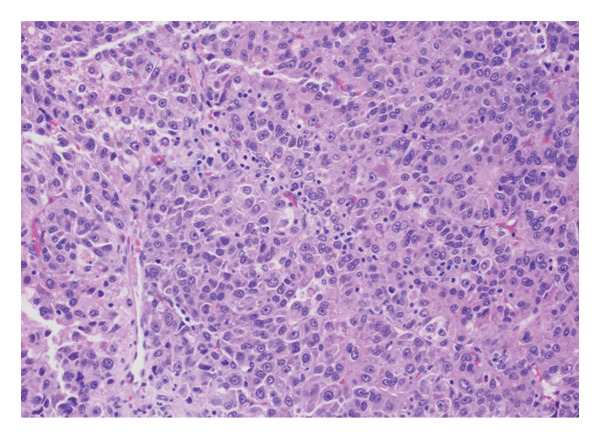
(a)

**Figure:**
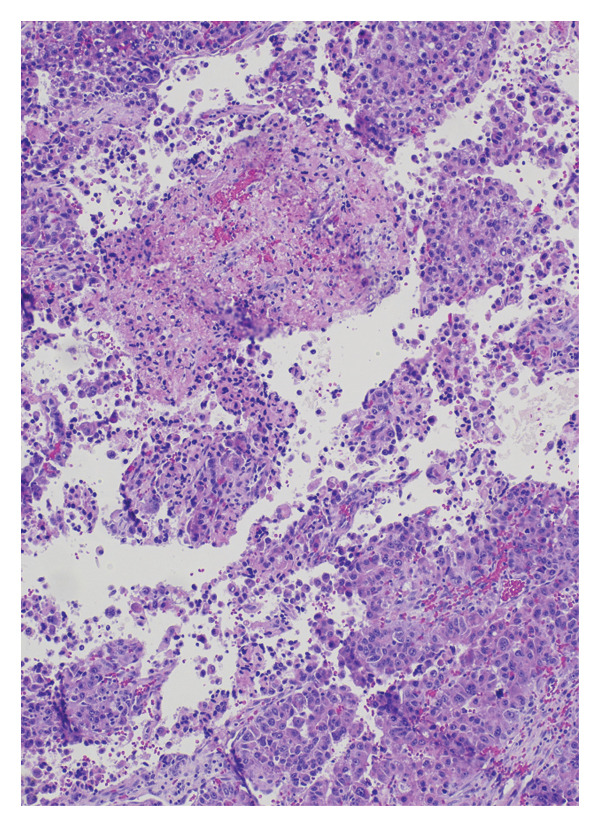
(b)

**Figure:**
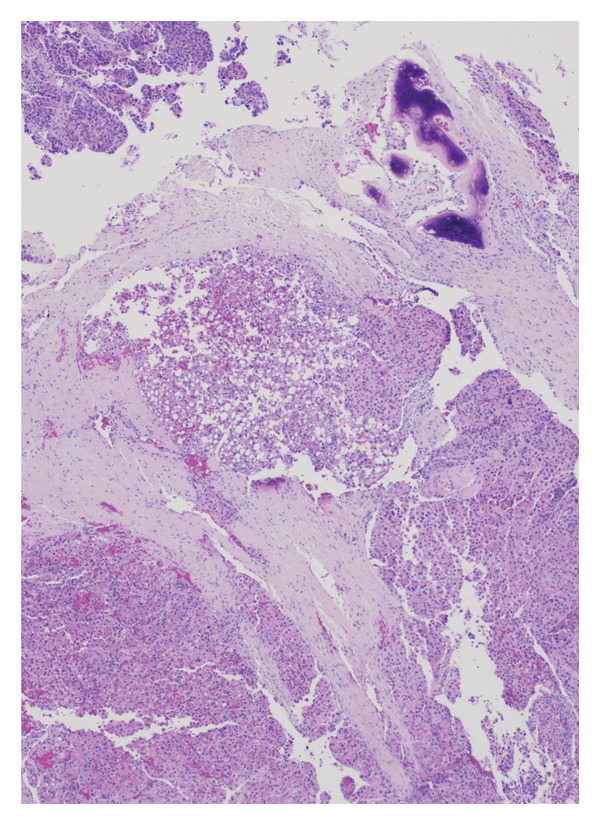
(c)

**Figure:**
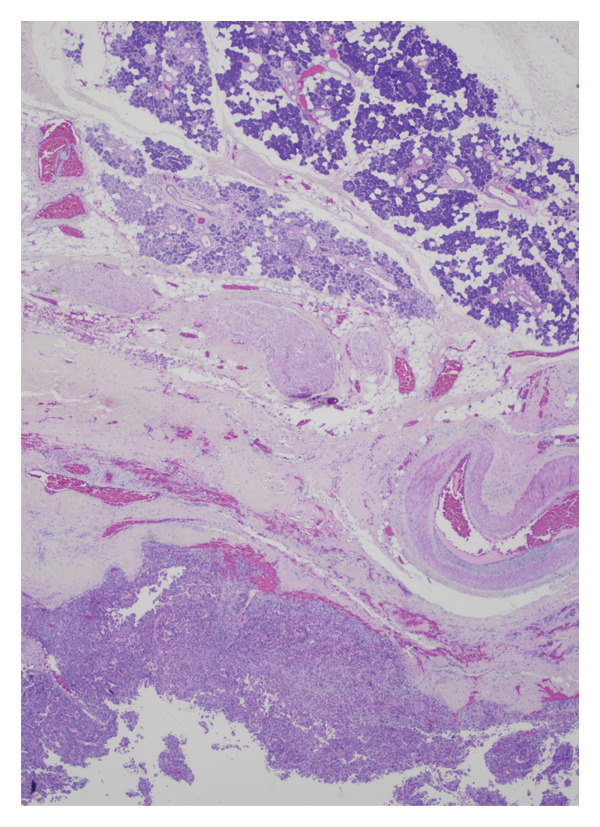
(d)

Three months later, he developed a recurrent 7‐cm mass with extension into the zygomatic arch, parotid space, and orbit. AFP rose to 235 ng/mL. PET‐CT revealed no distant metastasis. He received lenvatinib and stereotactic body radiation therapy (30 Gy in 5 fractions). Despite therapy, the disease progressed with bilateral pulmonary nodules and a T9 vertebral metastasis with epidural extension. AFP exceeded 600 ng/mL. He started cabozantinib, but the disease progressed. He entered hospice and died 13 months after diagnosis of metastatic recurrence.

## 3. Discussion

HCC recurrence after liver transplantation typically occurs within 2 years and most often involves the lungs, bones, or lymph nodes [1, 2]. Parotid involvement is infrequent, with only a few reports describing metastases to the head and neck region, most commonly the mandible, skull base, or maxilla [4, 6].

This patient met Milan criteria, had moderately differentiated pT2 disease, and remained disease‐free under standard surveillance. Nonetheless, recurrence occurred 31 months posttransplant, beyond typical surveillance windows and in an anatomical region not routinely imaged. AFP remained normal until symptom onset and rose only modestly at the time of diagnosis. This case illustrates the importance of multidisciplinary follow‐up to identify rare or atypical metastatic presentations.

The timing of HCC recurrence after liver transplantation carries important prognostic significance. Recurrence is typically classified as early (within 2 years) or late (beyond 2 years), representing distinct biological entities with different clinical outcomes. Early recurrence, occurring in approximately 60% of recurrent cases, is associated with significantly worse prognosis, with reported median survival times of 14–15 months and 5‐year survival rates of only 6%–7%. In contrast, late recurrence demonstrates substantially better outcomes, with median survival of 17–21 months and 5‐year survival rates of 64% [7]. Our patient developed recurrence at 31 months posttransplantation, classifying this as late recurrence. This timing is consistent with a potentially more indolent tumor biology, though the ultimate aggressive course and rapid progression once recurrence was identified underscores the therapeutic challenges inherent to HCC recurrence in the immunosuppressed transplant population. Late recurrence may reflect occult micrometastatic disease present at the time of transplantation that remained dormant under immunosuppression, or alternatively, de novo tumor development in the context of ongoing viral hepatitis in cases where the underlying etiology was viral cirrhosis. Understanding the distinction between early and late recurrence patterns may inform surveillance strategies and guide clinical decision‐making regarding the intensity and duration of posttransplant monitoring.

In addition, the workup of a new mass in the setting of HCC s/p transplant should be re‐evaluated. Two core needle biopsies were nondiagnostic and may have delayed diagnosis. Imaging suggested malignancy, and an earlier surgical biopsy could have expedited treatment. In well‐vascularized or necrotic lesions, especially in transplant recipients, physicians should consider early excisional biopsy when clinical suspicion remains high.

Immunosuppression likely influenced the aggressive disease course. The patient required multiple reoperations and remained on calcineurin inhibitors and mycophenolate throughout his posttransplant course. His early postoperative complications requiring multiple reoperations may have necessitated prolonged or intensified immunosuppression, potentially contributing to impaired tumor surveillance. Calcineurin inhibitors impair immune surveillance and activate prooncogenic pathways such as TGF‐β [8]. Although mTOR inhibitors may reduce recurrence via antiproliferative effects [9], the oncology team did not recommend the conversion. Checkpoint inhibitors were withheld due to the risk of graft rejection, which significantly limited systemic therapy options. Despite treatment with two tyrosine kinase inhibitors, the disease progressed rapidly, underscoring the therapeutic challenges of managing recurrent HCC in immunosuppressed patients.

The patient’s immunosuppression adhered to standard protocols: triple therapy (corticosteroids, tacrolimus, and mycophenolate) for 3.5 months, followed by dual therapy (tacrolimus plus mycophenolate). Tacrolimus trough levels remained within the therapeutic range (6.0 ng/mL at recurrence; target 4–8 ng/mL). However, the complicated early postoperative course, including multiple reoperations for bile leak and bleeding, MRSA bacteremia, and acute cellular rejection on Postoperative day 16, may have required more aggressive immunosuppression during the crucial first month, potentially compromising tumor immune surveillance. The detection of extrahepatic recurrence 31 months after transplant, despite favorable explant pathology (pT2, G2, and Milan criteria), suggests the presence of occult micrometastatic disease at the time of transplantation that went undetected by standard imaging.

This case underscores three insights: first, HCC can recur late and in atypical locations even with favorable pathology and appropriate surveillance; second, facial or parotid masses in liver transplant recipients warrant prompt oncologic evaluation, regardless of AFP levels; and third, core needle biopsy may be insufficient in certain soft tissue metastases and should not delay definitive diagnosis and management.

More broadly, this case necessitates re‐examination of fundamental assumptions in posttransplant HCC surveillance. As immunosuppressive protocols evolve and patient survival improves, we must consider whether current surveillance strategies adequately capture the full spectrum of recurrence patterns. Questions arise regarding optimal surveillance duration, the role of cross‐sectional imaging beyond the chest and abdomen, and the integration of novel biomarkers or liquid biopsy techniques. Furthermore, the therapeutic limitations highlighted here underscore the urgent need for HCC treatment strategies specifically designed for the immunosuppressed transplant population.

## 4. Conclusion

This case represents the first reported instance of HCC metastasizing to the parotid gland after liver transplantation. It highlights the limitations in current transplant oncology practice, particularly regarding the scope and duration of surveillance, the diagnostic strategies for atypical lesions, and the systemic treatment restrictions imposed by immunosuppression. Targeted improvements in these areas will be critical to optimizing long‐term outcomes for transplant recipients.

**Nomenclature**
AFP: Alpha‐fetoprotein
CMV: Cytomegalovirus
DCD: Donation after circulatory death
HCC: Hepatocellular carcinoma
LI‐RADS: Liver imaging reporting and data system
MELD: Model for End‐Stage Liver Disease
MRSA: Methicillin‐resistant *Staphylococcus aureus*
MRI: Magnetic resonance imaging
mTOR: Mechanistic target of rapamycin
PET‐CT: Positron emission tomography–computed tomography
RAI: Rejection activity index
TACE: Transarterial chemoembolization
TGF‐β: Transforming growth factor beta

## Conflicts of Interest

The authors declare no conflicts of interest.

## Funding

No external funding was received for this work.

## Data Availability

Data sharing is not applicable to this article as no datasets were generated or analyzed during the current study.
